# A Compendium of Risk and Needs Tools for Assessing Male Youths At-Risk to and/or Who Have Engaged in Sexually Abusive Behaviors

**DOI:** 10.5964/sotrap.8085

**Published:** 2022-11-02

**Authors:** Sandy Jung, Mackenzie L. Thomas

**Affiliations:** 1 Department of Psychology, MacEwan University, Edmonton, AB, Canada; Simon Fraser University, Burnaby, Canada

**Keywords:** sexual offending, perpetration, risk tools, youths, risk assessment

## Abstract

Using a standardized, validated risk assessment tool is an integral part of risk management and should be employed to evaluate a youth who is at risk to and/or has engaged in sexually abusive behaviors. Risk and needs tools are needed to inform critical decisions about the allocation of services and the areas that should be targeted in treatment and supervision. Although practitioners have a number of published tools to their avail, it is often less practical to discover the type of tool, where to access the tool, information regarding its psychometric properties, and how to access relevant training. This paper offers a brief compendium of youth-applied risk tools specific to male youths who are at risk to and/or who have engaged in sexually abusive behaviors; specifically, a description of the tool and its psychometric properties, along with where practitioners may access these tools and any relevant training in using these tools, are summarized. In light of the challenges that exist when assessing risk among youths, caveats and considerations are also explored.

Employing an appropriate risk assessment tool is integral for the assessment and treatment of individuals who have engaged in sexually abusive behaviors, as well as other violent behaviors (Hanson, 2009). Much is known about assessing sexual violence risk among adult males who have been charged for sexual offending (see Zara et al., 2020, for a review of adult-based measures), but the literature is somewhat limited when applied to youths (Viljoen et al., 2020). Despite the relatively limited empirical literature on risk assessment of youths who have been charged for sexual offending, there is a number of tools available, and there have been many published studies examining these tools (see Viljoen et al., 2020, for an overview of general, violence, and sexual risk assessment for youths). However, reviews of youth-applied tools specific to sexually abusive behaviors have been in short supply, including a summary of these tools, where practitioners may access these tools, and any relevant training in using these tools.

This article provides a brief compendium of risk assessment instruments developed to assess the sexual violence risk of male youths who are at risk to and/or have engaged in sexually abusive behaviors. Specifically, for the purpose of this paper, youths refer to adolescents whose ages may range from 12 to 18 years when they committed the sexually abusive behavior, although some researchers have used slightly different age ranges (see Murphy et al., 2016). Also, consistent with limited evidence supporting empirically validated risk assessments for female adults who have sexually abused (see Miller & Marshall, 2019; Vess, 2011), the data is even more limited for female youths. Therefore, this review will focus on instruments developed for assessing male youths. For each instrument reviewed, tool descriptions, psychometric properties, tool access and availability, and access to training, when available, are summarized.

Assessing risk is a necessary component of reducing harmful behaviors, and its importance is infused in the overarching principles of risk, need, and responsivity (RNR; Bonta & Andrews, 2017). These principles guide who should receive service and how much service, what risk factors interventions should target, and how services should be most effectively delivered. Of the RNR principles, the risk principle refers to matching the level of service that should be delivered to a youth based on a valid assessment of risk. The risk principle states that interventions are most effective with youth deemed to be at a higher risk of reoffending. The second principle, known as the need principle, suggests that effective treatment targets dynamic criminogenic needs, which can reduce the youth’s overall risk to reoffend. Finally, the responsivity principle states that practitioners should tailor interventions to suit the individual to be most effective. The RNR principles have been applied to youths more generally (e.g., Brogan et al., 2015; Wylie et al., 2019) and have been explored with youths who have sexually abused (ter Beek et al., 2018). It is generally agreed that employing a risk tool provides a reliable assessment of youths when development processes and contexts are constantly changing, and that staff employing such tools should be thoroughly trained on the use of the tool (Brogan et al., 2015).

A key goal of employing a risk tool is to ensure an evidence-based approach is used to manage risk. It is important to ensure that there is a direct pathway between risk assessment and risk management and that employing a risk tool is not merely a bureaucratic exercise (Viljoen et al., 2018). Identification of risk factors are necessary to effectively intervene in the cycle of abusive behavior, followed by treatment and other management and restrictive approaches, which are seen as part of supervision. Furthermore, ensuring protective factors are built upon and strengthened in the process of managing risk is equally important when working with a developing group, such as youths at risk. To avoid using intuitive approaches or making non-evidence-supported decisions, one must ensure that meaningful psychological risk factors are considered (Mann et al., 2010).

A challenge that professionals often face is deciding which tool to employ in their practice. McGrath et al. (2010) authored a report entitled *Current Practices and Emerging Trends in Sexual Abuser Management* that reported the results of a survey conducted in 2009 (also known as the Safer Society Press Survey). In their report, they provided the prevalence of risk tool use by 275 U.S. and 15 Canadian adolescent male programs. They found that for each country, the Juvenile Sex Offender Risk Assessment Protocol (J-SOAP-II; Prentky & Righthand, 2003) and Estimate of Risk of Adolescent Sexual Offence Recidivism (ERASOR 2.0; Worling & Curwen, 2001), respectively, were used by more treatment programs for youths at risk of and/or who have engaged in sexually abusive behaviors. This survey was conducted over ten years ago, and there has been no published update since then. Furthermore, since 2009, many other tools have emerged or have been further developed.

This review aimed to provide a current and practical overview of existing tools and guidelines used to assess the risk for sexually abusive behaviors with youths at risk to and/or who have engaged in harmful sexual behaviors. As a result, a comprehensive search was conducted in various databases (e.g., PsycINFO, ERIC, Google Scholar) and reference lists from various articles. Sources included those drawn from published (including peer- and non-peer-reviewed papers), unpublished (e.g., manuscripts, theses/dissertations), and website sources. The following provides a summary of each discovered tool, along with directions on how to access the tool, the manual for the tool, and relevant training necessary for practitioners to soundly use and apply the tool in their practice validly and reliably^1^1The weblinks and training information provided were active at the time of writing. However, the authors recognize that the digital footprint of these resources may change upon publication.. In addition, when psychometric properties are available, namely interrater reliability and construct and predictive validity, these properties are summarized. Of note, when predictive validity is reported, best practices recommend using statistical analyses, such as receiver operating area under the curve characteristics (AUC; Helmus & Babchishin, 2017). According to Rice and Harris (2005), AUCs of .56, .64, and .71 correspond to the beginning of small, moderate, and large effect sizes (which are equivalent to Cohen’s *d* of .20, .50, and .80).

The following sections categorize these tools into actuarial and structured professional judgment tools. These are subsequently followed by a section that provides an overview of risk tools not explicitly designed for those who are at risk and/or engage in sexually abusive behaviors but, more broadly, for youths who are at risk and/or have engaged in criminal behavior. Although intended to be exhaustive, the authors recognize there are many existing tools that may not be published or available in English at the time of writing. Moreover, most tools described are primarily intended for use with male youths, with limited to no application to female youths. It is important to note that the focus of this review does not include children with sexual behavior problems (CSBP). The assessment and treatment of CSBP is known to differ from the assessment and treatment of adolescents who have engaged in sexually abusive behaviors, and therefore, a discussion of assessment and prevention efforts with CSBP would stretch beyond the scope of this paper (see Chaffin et al., 2006; DeLago et al., 2020; Miccio-Fonseca, 2020; for further discussion).

## Actuarial Risk Tools

Actuarial risk tools are those that rely on empirically supported risk factors and specific criteria to determine an individual’s risk categorization (Hanson, 2009). They are developed by selecting and combining risk factors that have been empirically or statistically associated with reoffending. In this approach, the role of professional judgment is limited, so a total score on the measure can be associated with outcome probabilities. The main advantages of using actuarial tools are the optimization of prediction and the more objective nature of scoring items. By statistically finding the best combination of factors that discriminate between groups who reoffend and those who do not, actuarial approaches are often seen as the most accurate approach (Hanson, 2009). Also, clear operational definitions of items on these tools and the mechanical nature of these operationalizations are less impacted by subjective or professional judgment (e.g., Harris et al., 2002). However, there are disadvantages of actuarial tools. One of the main criticisms is that risk factors that comprise these scales are not necessarily linked to nor guided by theory. Additionally, there may be relevant considerations not included in the tool. Actuarial risk tools for sexually abusive youths are described in the following subsections and listed in alphabetical order.

### Juvenile Risk Assessment Scale

The Juvenile Risk Assessment Scale (JRAS) is a 14-item scale designed to assess the risk of sexual recidivism among male adolescents who have been convicted of a sexual offence (Hiscox et al., 2007). The tool was originally developed in response to a New Jersey Supreme Court decision that the Attorney General must develop a risk assessment scale specific to juvenile sex offenders. The intention behind this decision was to use such a tool to assess risk reliably, in order to make decisions on whether to apply New Jersey's community notification law (Hiscox et al., 2007). The JRAS (adapted from the adult version called the Registrant Risk Assessment Scale or RRAS) is intended for use with male youths between 12 and 19 years of age. The measure contains scales for nine static and five dynamic items and is divided into the three broad areas of sex offence history (8 items; e.g., degree of force), antisocial behavior (2 items; e.g., substance abuse), and environmental characteristics (4 items; e.g., response to sex offender treatment; for items see Table 1). The 14 items are scored on a 3-point scale as low, moderate, or high, and the overall risk of recidivism is labelled as low (score of 0-9), medium (10-19), or high (20-28). The original composition of the JRAS has not changed since its development. The coding manual is available at no cost^2^2https://www.nj.gov/oag/dcj/megan/jras-manual-scale-606.pdf and outlines the specific coding criteria for each item. There are currently no official or ongoing training programs on the JRAS.

**Table 1:** Items or Risk Domains on Actuarial Tools, JRAS and JSORRAT-II

JRAS	JSORRAT-II
Degree of force Degree of contact Age of victim Victim selection Number of offenses/victims Duration of offensive behavior Length of time since last offense (while at risk) Victim gender History of antisocial acts Substance abuse Response to sex offender treatment Sex offender specific therapy Residential support Employment/educational stability	Number of juvenile sexual offense adjudications Number of victims in charged sexual offenses Sexual offending history duration Sexual offenses while under supervision Felony-level (“hands-on”) sexual offenses in public Use of deception or grooming in a charged sexual offense Completed prior sexual offender specific treatment Number of victimizations of “hands-on” sexual abuse Number of victimizations of physical abuse Special education placements Number of periods with school discipline problems Number of adjudications for non-sexual offenses

*Note.* The MEGA*♪* and the AIM3 are not publicly available (coding manuals are available for purchase).

The JRAS has very limited empirical support. Hiscox et al. (2007) found that the tool is a moderate predictor of sexual recidivism (AUC = .656). However, other published research suggests the JRAS does not significantly predict sexual, non-sexual violent, or general recidivism (Caldwell et al., 2008; Hempel et al., 2013). Although lacking in predictive validity, the JRAS does have moderate interrater reliability (*r* = .66; Hiscox et al., 2007).

### Juvenile Sexual Offense Recidivism Risk Assessment Tool – II

The Juvenile Sexual Offense Recidivism Risk Assessment Tool – II (JSORRAT–II) is a 12-item measure designed to assess the risk of juvenile sexual recidivism at the time of their index sexual offence (Epperson et al., 2006). It is used with male youths between 12 and 18 years of age. The tool is comprised solely of static items and addresses sex offence history, victimization of the youth evaluee, environmental risk factors, and non-sex offence history, and the scores range from 0 to 20 (see Table 1 for list of items). Although the tool is in its second iteration, the items have not changed since the second version was released. Comprehensive instructions for coding each specific item can be found in the coding manual, which is available at no cost^3^3http://www.watsa.org/Resources/Documents/2.Epperson%20JSORRAT-II%20Scoring%20Guide.pdf. Training on administering the JSORRAT-II is required and is available through The Global Institute of Forensic Research, Inc.^4^4https://gifrinc.com/jsorrat-ii/. In addition, there are a number of certified trainers who offer training^5^5https://saratso.org/index.cfm?pid=1357.

The JSORRAT-II has some empirical support. It has strong interrater reliability (intraclass coefficient [ICC] = .89; Viljoen et al., 2008). According to a meta-analysis by Viljoen et al. (2012), the JSORRAT-II is a moderate predictor of sexual recidivism, AUC = .64, 95% CI [.54, .74]; *k* = 7. Similar results were found by Epperson and Ralston (2015), who determined that the measure moderately predicted sexual recidivism among justice-involved juveniles, AUC = .65, 95% CI [.59, .72]. However, some studies have found the tool has low predictive power, AUC = .57, 95% CI [.43, .72] (Rasmussen, 2018). Confounding factors may also impact its accuracy as a risk tool. Ralston et al. (2016) found that, although the tool performed well for those aged 11 to 15 years, it did not significantly predict recidivism among those aged 16 to 17. Furthermore, when individuals whose only violent offence was sexual were removed from the sample, the predictive validity of the tool became nonsignificant, AUC = .57, 95% CI [.44, .66].

### Multiplex Empirically Guided Inventory of Ecological Aggregates for Assessing Sexually Abusive Children and Adolescents

The Multiplex Empirically Guided Inventory of Ecological Aggregates for Assessing Sexually Abusive Children and Adolescents (MEGA♪) is a tool intended to measure the risk of sexually abusive behaviors in youths (male, female, or non-binary) who are between the ages of 4 to 19 years old (Miccio-Fonseca, 2012). The tool is intended for use with youths who have been adjudicated or non-adjudicated for their behavior, and the authors indicate it can be used with female youths, ethnic minorities, individuals with psychiatric disorders, and those with low intellectual functioning (Miccio-Fonseca, 2013, 2016). The MEGA♪ comprises four scales: The Risk Scale, the Protective Scale, the Estrangement Scale, and the Historic Correlative Scale. The measure simultaneously assesses the risk level for coarse sexual behaviors (i.e., crude, indecent, outside social norms; e.g., crude sexual gestures, vulgar sexual comments, looking up skirts, looking over bathroom stall) and sexually abusive behaviors, with items being scored as either yes or no. The overall risk of recidivism is coded as low, moderate, high, and very high, and a comprehensive risk assessment report that details risk factors, protective factors, and changes in risk over time is generated. With re-assessments recommended in six-month intervals, the MEGA♪ can serve as a useful outcome measure of treatment progress. The tool has not changed since its original development. Information regarding training workshops to certify practitioners to administer the tool can be found on the official MEGA♪ website^6^6https://www.mega-miccio-fonseca.com/sexual-abuse.html. Once training is completed, instructions will be provided on how to purchase the MEGA♪ risk reports.

The MEGA♪ is well-supported empirically. The measure has strong interrater reliability, with the tool achieving over 98% score agreement when assessed by multiple individuals trained to administer the MEGA♪ (Miccio-Fonseca, 2013). In terms of its use as a predictor of sexual recidivism, the tool has demonstrated moderate predictive accuracy, AUC = .67, 95% CI [.52, .82] (Rasmussen, 2018).

## Structured Professional Judgement Tools

Structured Professional Judgement (SPJ) tools rely on the expertise of the rater to form an overall judgment of risk, and the risk categorization or score derived from adding the number of risk factors present are not linked actuarially to recidivism outcomes (Hanson, 2009). The SPJ approach relies on theory- or evidence-based guidelines to systematize the exercise of discretion. It involves a series of stages that include gathering relevant information in order to determine the presence of relevant risk factors, using discretion to weigh and combine risk factors to characterize the potential for violence, and planning a course of action to mitigate violence (Hart et al., 2016). The composition of risk factors in SPJ tools is often based on comprehensive reviews of the literature that have identified variables most frequently or strongly associated with the harmful outcome of interest (Logan, 2016).

Similar to actuarial tools, there are advantages and disadvantages of SPJ approaches. The advantages of employing SPJ tools are that they are intended to broaden the approach to risk assessment beyond risk formulation and entail identifying treatment targets and strengths of the assessed individual, planning for precipitating factors, and anticipating future scenarios where the individual would be at risk (Logan, 2016). One disadvantage is that although risk factors are specified in advance, the overall assessment of risk is based on professional judgment with no quantification of scores or totalling of factors (Hanson, 2009); this can introduce greater subjectivity or biases. As these measures rely on professional judgement, there are no cut-off scores to determine an individual’s level of risk. Hence, another issue that has been raised is that the risk judgment, which typically includes low, moderate, and high, is not linked to estimated outcome probabilities (Hanson, 2009). The following subsections summarize SPJ tools applied to youth who engage in sexually abusive behavior in alphabetical order.

### Assessment, Intervention, Moving On – Version 3

The Assessment, Intervention, Moving On – Version 3 (AIM3) assessment framework consists of 25 items intended to assess the risk of recidivism, the required level of supervision, and factors to target in treatment (Henniker et al., 2002). The instrument is primarily intended for use with male youths between the ages of 12 and 18 years old with a history of sexual abuse perpetration. Application to female youths, individuals with learning disabilities, and ethnic minorities may be done on a case-by-case basis with special consideration of how behaviors and attitudes of these youths may present differently. The developers specifically note that the AIM3 may be used to assess risk for contact offences, as well as technology-assisted sexual offences (e.g., downloading indecent images of children), and instances of sibling abuse. Historical risk factors are considered for their relevance to the youth at the present time, but the AIM3 is made up of predominantly dynamic items. The measure contains five domains of items which include sexual behaviors, non-sexual behaviors, developmental factors, situational factors, and self-regulation competencies. The score of each item ranges from zero to four, with a possible total of 20 points in each domain. Furthermore, the scores on each domain indicate the level of need or risk in a particular area. However, it is up to the practitioner's discretion to determine the youth's overall risk level. It is not clear if the tool has been updated since its original development since no coding manual is publicly available at the current time. However, training can be purchased and accessed on the AIM Project website^7^7https://aimproject.org.uk/portfolio-item/organised-training/, whereupon access to the materials would be provided.

The third version of the AIM assessment framework does not have any published empirical support at the time of writing. However, current studies are underway to assess the effectiveness of the AIM3 (Christina Adamson, personal communication, Jan 4, 2022). Research looking into the psychometric properties of the previous version of the measure indicates that two subscales, in particular, the concerns scale, AUC = .98, 95% CI [.98, 1.00], and the strengths scale, AUC = .94, 95% CI [.89, 1.00], have strong predictive validity for sexual reoffending (Griffin et al., 2008); however, the study included a small sample of 70 with only 7 recidivists and the confidence intervals may be inflated as a result. Griffin et al. (2008) suggested that the previous second version of the AIM framework had good interrater reliability. Thirty-six items had kappa coefficients above 0.75, twenty had a kappa between 0.6 and 0.75, and only two had a kappa below 0.6.

The AIM3 has been adapted for younger children. The adapted AIM3 framework for use with younger children also includes those with learning disabilities. Two approaches are offered depending on the child's age. For children under seven years old, pattern mapping is utilized (i.e., a visual framework exercise that captures key life events and sexual behaviors to identify what has led to the behavior, who is targeted, what methods are used to engage with victims, where and when the behavior is happening, the meaning of the behavior, and how motivated the child is to change the behavior). For children between the ages of 8 and 12 years, a dynamic risk assessment model is used. The dynamic nature of the assessment allows it to measure changes in sexual behaviors; thus, it can be useful for monitoring the efficacy of an intervention. At this time, there are no empirical studies on these adapted versions of the AIM3 tool.

### Estimate of Risk of Adolescent Sexual Offense Recidivism

The Estimate of Risk of Adolescent Sexual Offence Recidivism, version 2.0 (ERASOR 2.0) is a tool intended to measure the short-term risk of sexual recidivism in male youths between 12 and 18 years of age who have previously committed sexual offences (Worling & Curwen, 2001). To target criminogenic areas in order to help tailor interventions, the risk tool comprises a single-scale instrument with 25 risk factors. These risk factors are categorized into five subtypes which include Sexual Interests, Attitudes and Behaviours, Historical Sexual Assaults, Psychosocial Functioning, Family/Environmental Functioning, and Treatment (see Table 2). All risk factors are coded as either Present, Possibly/Present, Not Present, or Unknown. Due to the nature of the measure as a structured professional judgment tool, there are no cut-off scores to determine an individual’s level of risk. The coding manual, which is no longer available by the developer/author, but publicly available at no cost through various other personal and government websites^8^8Websites with the ERASOR 2.0 coding manual and form, posted on government and personal websites, which are publicly available: https://djj.ky.gov/800%20Policy%20Manual/ERASOR%202.0%209-1-20.pdf/https://grahamwatson.ca/resources/erasor_2.0_10-page_coding_form.pdf, outlines the specific coding criteria and research and clinical support for each factor. Only the second iteration of the tool is available, and research has focused on this second version. Training on administering the ERASOR 2.0 was previously available through the developer (J. Worling), but currently, no training is available on the tool, despite its availability and continued use.

**Table 2:** Items or Risk Domains on Structured Professional Judgment Tools, ERASOR 2.0, GAIN, J-RAT, and J-SOAP-II

ERASOR 2.0 (25 items; 5 subtypes)	GAIN (34 items; 6 risk domains)	J-RAT (97 items; 12 risk domains)	J-SOAP-II (28 items; 4 risk domains)
Sexual interests, attitudes and behaviours Historical sexual assaults Psychosocial functioning Family/environmental functioning Treatment	Sexual behavior characteristics Victimization experiences Violence and control Personal and interpersonal characteristics Family characteristics Intervention	History of sexually abusive behavior History of non-sexual antisocial behaviors Responsibility Relationships Cognitive capacity and ability Social skills Developmental adversity/trauma Personal characteristics and qualities Psychiatric comorbidity and treatment Substance abuse Family factors Environmental conditions	Sexual drive/sexual preoccupation Impulsive/antisocial behavior Clinical/treatment Community adjustment

The ERASOR 2.0 is well-supported empirically. When the items are mechanically totalled (which is not the intended approach by the tool developer), the tool has been demonstrated to be a strong predictor of sexual, AUC = .71, 95% CI [.62, .80] and non-sexual recidivism, AUC = .71, 95% CI [.69, .79] (Rajlic & Gretton, 2010). It is notable that Barra et al. (2018) found that clinical judgment ratings (which is the way the developer intended the tool to be used) significantly predicted sexual reoffending with a large effect size, AUC = .76, 95% CI [.67, .85]. However, a meta-analysis (Viljoen et al., 2012) revealed a moderate effect for sexual recidivism, AUC = .66, 95% CI [.61, .72]; *k* = 10, and small effect for non-sexual recidivism, AUC = .59, 95% CI [.50, .67]; *k* = 7, when items were mechanically totalled, with similar effects when clinical judgment ratings were examined (sexual recidivism, AUC = .66, 95% CI [.60, .71]; *k* = 9; non-sexual recidivism, AUC = .59, 95% CI [ .51, .68]; *k* = 6). When applied to juvenile males with sexual offences, the measure is best-suited to predict sexual recidivism within 0.5 to 3 years (Barra et al., 2018). Additionally, it was shown to have strong interrater reliability (ICC = .88; Worling et al., 2012). There is no current data examining the use of the ERASOR 2.0 with youths who have been diagnosed with mental disorders. However, there is some support for its use cross-culturally (e.g., Chu et al. [2012] reported AUC = .74, 95% CI [.61, .88], in Singapore).

### Guided Assessment of Intervention Needs

The Guided Assessment of Intervention Needs (GAIN), formerly known as the Assessing Risk to Repeat Sexual Behaviour Problems – Version 2.1 (AR-RSBP), is a 34-item measure intended to assess the likelihood a child under the age of 12 years will re-engage in sexually abusive behaviors (Curwen, 2011). The measure is comprised of both static and dynamic items that are organized into six domains, which include sexual behavior characteristics, victimization experiences, violence and control, personal and interpersonal characteristics, family characteristics, and intervention (refer to Table 2). Modelled off the ERASOR 2.0 (Worling & Curwen, 2001), the GAIN relies on empirical evidence on risk factors and professional judgment. As such, no cut-off scores are provided to determine the categorization of risk and are best suited to be used in conjunction with other measures for assessment. Training is not required to administer the GAIN, but practitioners are encouraged to have an understanding of child developmental psychology (Curwen, 2011). There are currently no published studies assessing the psychometric properties of the GAIN; therefore, very little is known about this tool.

### Juvenile Risk Assessment Tool – Version 4

The Juvenile Risk Assessment Tool (J-RAT) is a tool meant to assess the risk for sexual recidivism in adolescent males aged 12 to 18 years old who have or are alleged to have engaged in sexually abusive behavior (Rich, 2017a). The measure consists of 97 items grouped under 12 risk domains, each representing a risk factor frequently cited in the research literature (see Table 2). The 97 items include 24 protective factor items. There are also adaptations of the J-RAT designed explicitly for other populations: The Intellectual Disability Juvenile Risk Assessment Tool (ID/J-RAT*) was created for use with lower functioning adolescents, and the Latency Age-Sexual Adjustment and Assessment Tool (LA-SAAT) for use with males aged 8 to 13 years old (Rich, 2017b; Rich, 2019). Training is not required to administer the J-RAT, but practitioners are encouraged to have training and experience assessing risk among youths within a mental health context. Given that the J-RAT is not statistically derived and the items are not quantified (i.e., items are not scored and totaled), the authors intended for the J-RAT to be used as an organized method for the clinical assessment of risk for sexual recidivism and, therefore, there are no published studies on the psychometric properties (Phil Rich, personal communication, October 19, 2021). Open access to the full measures and coding manuals can be found on Dr. Phil Rich’s official website at no cost^9^9http://www.philrich.net/risk-assessment-instruments.html.

### Juvenile Sex Offender Risk Assessment Protocol

The Juvenile Sex Offender Risk Assessment Protocol (J-SOAP-II) aims to assess the risk for sexual recidivism in male youths between 12 to 18 years old who have been adjudicated for a sexual offence or have a history of sexually abusive behaviors (Prentky & Righthand, 2003). The J-SOAP-II was initially developed based on a systematic review of risk factors that have been identified in the research literature as being associated with sexual and criminal offending, and the current version comprises 28 items. These items are categorized under four domains, which include Sexual Drive/Sexual Preoccupation, Impulsive/Antisocial Behavior, Clinical/Treatment, and Community Adjustment (refer to Table 2). Each item is scored on a 3-point Likert-type scale with responses ranging from zero to two depending on how present the factor is. Further information about scoring the J-SOAP-II can be found in the coding manual, which is publicly available at no cost^10^10https://www.ojp.gov/pdffiles1/ojjdp/202316.pdf. Training on administering the J-SOAP-II is available through the combined training via the Association for the Treatment of Sexual Abusers Master Class learning series^11^11https://atsa-training.com/course/risk-assessment-with-adolescents-who-have-sexually-offended/.

The J-SOAP-II is well-supported empirically when the mechanical total of the items and subscale items have been examined. It has been demonstrated to be a moderate predictor of sexual, AUC = .67, 95% CI [.59, .75]; *k* = 9, and non-sexual recidivism, AUC = .66, 95% CI [.57, .75] (Viljoen et al., 2012); *k* = 7, when mechanically totalled. Aebi et al. (2011) found that, in particular, sexual recidivism was significantly predicted by the antisocial (AUC = .74) and adjustment (AUC = .74) subscales. It is also shown to have strong inter-rater reliability (ICC = .77; Chu et al., 2012). The J-SOAP-II has not been indicated for use with youths with cognitive limitations. Furthermore, some results suggest it may be slightly less accurate a predictor for the risk of sexual recidivism in youths under 15 years (Viljoen et al., 2008). However, there is some support for its use among ethnic minorities, as seen in Martinez et al.’s study (2007) that included a predominantly Latino and African American sample (50% and 28%, respectively), AUC = .78, 95% CI [.66, .91].

### Protective + Risk Observations for Eliminating Sexual Offense Recidivism

The Protective + Risk Observations for Eliminating Sexual Offense Recidivism (PROFESOR) aims to identify risk and protective factors for sexual reoffending. It is appropriate for use in male or female adolescents aged 12 to 25 who have a history of sexual offence perpetration (Worling, 2017). Each scale on the 20-item tool is scored as being protective, neutral, or a risk, and final classifications range from Category 1 (*Predominantly Protective*) to Category 5 (*Predominantly Risk*; see Table 3 for a list of items). The measure contains solely dynamic risk factors, making it a useful tool to assess change over time or following an intervention. Information regarding training, access to the PROFESOR, and the coding manual can all be found on the official PROFESOR^12^12https://www.profesor.ca/ website at no cost.

**Table 3:** Items on Structured Professional Judgment Tools, PROFESOR and YNPS

PROFESOR (20 items)	YNPS (22 items)
Hope regarding healthy sexual future Sexual environment Sexual beliefs and attitudes Sexual interests - Focus Sexual interests - Frequency Knowledge of laws and procedures for respectful sexual relationships Knowledge of consequences of sexual offending Strategies to prevent sexual offending Compassion for others General values and attitudes Self-regulation Problem solving Responsivity to guidance and support Self-esteem Intimacy and friendship Relationship with parent/caregiver Parent/caregiver practices Engagement in school/work Engagement in organized leisure activity Living arrangement	Understanding appropriate sexual behavior Understanding the consequences of sexual abuse Sexual thoughts - Frequency Sexual interests - Age and consent Sexual attitudes and beliefs Sexual behavior management Compassion for others Relationships with peers Emotion management Social skills Self-confidence School and work commitment Use of unstructured time Nonsexual behavior attitudes and beliefs Nonsexual behavior management Client view of primary caregiver relationship Client view of supportive adult relationships Family functioning Living situation - Safety and stability Involvement in community resources Mental health management Participation in interventions

Unlike other measures, the PROFESOR is not designed to predict the risk of future reoffending. However, it can assist in identifying targets for interventions and thus reduce sexual recidivism through addressing these identified criminogenic needs. No empirical research currently exists on the PROFESOR. However, the author notes that a large-scale study is being conducted in the United States looking at interrater reliability, concurrent validity, and predictive validity, and results may be available in 2022 (James Worling, personal communication, December 31, 2021).

### Youth Needs and Progress Scale

The Youth Needs and Progress Development and Implementation Project was initiated in 2016 in order to address the limitations of existing risk tools to assess youths (e.g., developmental immaturity, absence of protective factors, low base rates), and one of its goals was to develop an evidence-informed treatment needs and progress scale (Kang et al., 2019; Prentky et al., 2020). The project led to the development of the Youth Needs and Progress Scale (YNPS; predecessor called Treatment Needs and Progress Scale, TNPS), which comprises 22 dynamic risk and protective factors, along with a few historical items, and is intended to identify risk-relevant intervention needs and track progress among youths and young adults ages 12 to 25 who have engaged in abusive sexual behavior (Righthand et al., 2020; for items see Table 3). The YNPS was originally scored via a software program but the authors noted that this was no longer an option once the project expired (Kang et al., 2019). The YNPS is available at no cost on the National Center on the Sexual Behavior of Youth website^13^13https://www.ncsby.org/sites/default/files/Youth%20Needs%20and%20Progress%20Scale-%20July%207,%202020.pdf. At the time of writing, no formal training or list of certified trainers was available.

There have been some researchers who have criticized the development (see Miccio-Fonseca, 2021) and applicability of the tool (see Rasmussen & Fagundes, 2022). For instance, it was noted that in its development, some tools were excluded from the original review of existing tools, which included the MEGA♪ (Miccio-Fonseca, 2021). Others have criticized the difficulty of applying the YNPS in practice and highlighted that the ambiguity in scoring items on a 5-point scale and poor operationalized definitions prohibited reliable and meaningful completion of the tool, and the process of calculating a total score was time-consuming (Rasmussen & Fagundes, 2022). There is no published empirical research examining the reliability and validity of the YNPS.

## Other Risk Tools Not Designed for Youths Who Engage in Sexually Abusive Behaviors

The above-described tools were specifically designed to assess sexually abusive behaviors or the risk of sexual recidivism in youth. However, in many cases of sexually abusive youths, other tools may be useful to assess violent or general risk for reoffending. They may also serve as supplemental measures for practitioners conducting a risk assessment. The following describes a non-exhaustive list of relevant risk assessment tools.

### Structured Assessment of Protective Factors for Violence Risk – Youth Version

The Structured Assessment of Protective Factors for Violence Risk – Youth Version (SAPROF-YV) is a violence risk assessment tool used to assess 16 dynamic protective factors for violence risk in adolescents and designed as an SPJ tool (de Vries Robbé et al., 2015). As this measure only addresses protective factors and not risk, it is not meant to be used as a risk tool, per se. Instead, it is intended to be used in conjunction with risk-focused structured professional judgement assessment or actuarial risk tools. The protective factors are categorized into four domains: Resilience, motivation, supports, and situational factors (for items, refer to Table 4). Each item is scored on a 7-point scale, which ranges from -2 to +2, and the practitioner indicates up to three critical factors and goals to assist in planning risk-reducing interventions. Although the authors note that it is not a requirement to receive training to administer the SAPROF-YV, training is available via the official SAPROF website and includes instructions on how to purchase the coding manual and coding sheet for the SAPROF-YV^14^14https://www.saprof.com.

**Table 4:** Items or Risk Domains on Other Tools Not Designed for Youths Who Engage in Sexually Abusive Behaviors, SAPROF-YV, SAVRY, and YLS/CMI 2.0

SAPROF-YV (16 items)	SAVRY (24 risk items and 6 protective factors)	YLS/CMI 2.0 (42 items; 8 risk domains)
Social competence Coping Self-control Perseverance Future orientation Motivation for treatment Attitudes towards agreements and conditions Medication School/work Leisure activities Parents/guardians Peers Other supportive relationships Pedagogical climate Professional care Court order	History of violence History of nonviolent offending Early initiation of violence Past supervision/intervention failures History of self-harm or suicide attempts Exposure to violence in the home Childhood history of maltreatment Parental/guardian criminality Early caregiver disruption Poor school achievement Peer delinquency Peer rejection Stress and poor coping Poor parental management Lack of personal/social support Community disorganization Negative attitudes Risk taking/impulsivity Substance-use difficulties Anger management problems Low empathy/remorse Attention deficit/hyperactivity difficulties Poor compliance Low interest/commitment to school Prosocial involvement Strong social support Strong attachment and bonds Positive attitude toward intervention and authority Strong commitment to school Resilient personality traits	Prior and current offenses/ dispositions Education/ employment Substance abuse Personality/behavior Family circumstances/ parenting Peer relations Leisure/recreation Attitudes/orientation

*Note.* The VRS-YV and the YLS/CMI 2.0 are not publicly available (coding manuals are available for purchase).

The SAPROF-YV has limited support. Preliminary findings from Zeng et al. (2015) found that the total domain score did not predict sexual desistance, AUC = .48, 95% CI [.28, .69]. These results were replicated in Koh et al.’s study (2022), which reported that the SAPROF-YV was not a strong predictor of general, violent, and non-violent reoffending after one year (AUC = .59, .59, .58, respectively) or after three years (AUC = .62, .59, .59, respectively). Furthermore, when they compared the SAPROF-YV with other youth violence risk tools, Koh et al. (2022) concluded that the SAPROF-YV does not add any significant value to the predictive validity when used in conjunction with each other. In terms of convergent and discriminant validity, an unpublished study by Bhanwer (2016) concluded that the SAPROF-YV displayed good congruence with the SAVRY (discussed below).

### Structured Assessment of Violence Risk in Youth

The Structured Assessment of Violence Risk in Youth (SAVRY) is an SPJ risk assessment tool used to make recommendations about the nature and degree of risk that an adolescent between the ages of 12 and 18 years may pose for future violence (Borum et al., 2006). It comprises 24 risk factors, including historical risk factors, social/contextual risk factors, individual/clinical factors, and six protective factors (see Table 4 for items). Notably, the SAVRY does not contain numerical values and does not offer specified cut-off scores, so it is primarily used to identify risk factors to target in interventions. Information about the specific items on the SAVRY and how to code them is available for purchase^15^15https://www.parinc.com/Products/Pkey/390. Furthermore, comprehensive training on administering the tool is available through The Global Institute of Forensic Research, Inc.^16^16https://gifrinc.com/savry/.

The SAVRY is empirically well-supported. It has been demonstrated to be a moderate predictor of violent recidivism after one year, AUC = .66, 95% CI [.54, .77], and after three years, AUC = .77, 95% CI [.67, .87] (Meyers & Schmidt, 2008), suggesting that the tool may perform better over a longer period than in the immediate future. Consistent with these findings, the SAVRY was also shown to predict adolescent violence after a long-term follow-up of four to seven years (Sijtsema et al., 2015). The tool applies to various populations, including females, AUC = .80, 95% CI [.59, 1.0], although there are higher incidences of false positives in females who offended than in males (Lodewijks et al., 2008; Meyers & Schmidt, 2008). Studies have also been done on adolescents with mental disorders, where the SAVRY was the most accurate predictor of violent offending and non-violent criminal conduct, as well as with ethnic minorities, including Indigenous Canadian youth at one-year follow-up, AUC = .64, 95% CI [.44, .83] and at three-year follow-up, AUC = .84, 95% CI [.69, .98] (Gammelgård et al., 2015; Meyers & Schmidt, 2008). Additionally, the SAVRY demonstrated excellent inter-rater reliability in relation to the total score (ICC = .97) and the risk rating (i.e., low, moderate, or high, determined by factors and other relevant information; ICC = .88; Dolan & Rennie, 2008). In terms of its practical application, it was found that practitioners who included the SAVRY measure had significantly better case plans for youth on probation (Viljoen et al., 2019).

### Violence Risk Scale—Youth Version

The Violence Risk Scale**—**Youth Version (VRS-YV) is a 23-item tool designed to assess risk, criminogenic needs, and change for adolescents within the youth justice system who have been identified as being at risk of committing violent offences (Wong et al., 2004-2011). Although the VRS-YV was developed in a similar way in how SPJ tools are designed (i.e., review of the literature to identify factors), it is modeled after its adult predecessor, the Violence Risk Scale (Wong & Gordon, 1999-2003), which is an actuarial tool. Consisting of both static and dynamic items, the scores of items on the VRS-YV range from zero to three, with items scored a two or three being considered important criminogenic targets for interventions and a higher total score on the measure indicating a greater risk of violent recidivism. While training is not required to administer the VRS-YV, practitioners are encouraged to have expertise and understanding of child development. Further information on accessing the measure, purchasing the coding manual, and obtaining training can be found on the Psynergy website^17^17https://psynergy.ca/.

The VRS-YV is empirically well supported. The measure has demonstrated high inter-rater reliability (ICC = .90) for the composite pre-treatment total, and can significantly predict the risk of violent, AUC = .77, 95% CI [.70, .85], nonviolent, AUC = .72, 95% CI [.63, .81], and general recidivism, AUC = .73, 95% CI [.64, .82] (Stockdale et al., 2014). Furthermore, there is some support for its use with females, AUC = .66, 95% CI [.53, .78] and Indigenous youth, AUC = .72, 95% CI [.62, .83].

### Youth Level of Service/Case Management Inventory 2.0

The Youth Level of Service/Case Management Inventory 2.0 (YLS/CMI 2.0) is a standardized instrument for individuals ages 12 to 18 years to assess the risk of recidivism, the need for correctional programs to reduce recidivism, and responsivity factors that impact case plan goals (Hoge & Andrews, 2002). The tool is most similar to actuarial risk tools and includes a 42-item checklist that produces a detailed survey of youth risk and needs factors to formulate a case plan. There are eight domains of risk factors on the YLS/CMI 2.0 that result in risk classification of low, moderate, high, or very high. The measure is available for purchase^18^18https://cad.storefront.mhs.com/collections/yls-cmi-2-0, and training is available through Multi-Health Systems^19^19https://www.gifrinc.com/yls-cmi/.

The YLS/CMI 2.0 has excellent inter-rater reliability (ICC =.95; Rennie & Dolan, 2010). Furthermore, the tool predicts recidivism with small effects among male, AUC = .62, 95% CI [.59, .65], and female youths, AUC = .57, 95% CI [.51, .62] between the ages of 12 and 18 years old (Anderson et al., 2016). Onifade et al. (2009) found that it can be a useful predictor for ethnically diverse populations, as no significant difference was found in the YLS/CMI 2.0’s predictive validity when applied to Caucasian youth (AUC = .66) versus African American youth (AUC = .63). Some research also suggests that the measure applies to individuals with psychiatric disorders. Rennie and Dolan (2010) found that in a sample of male youth diagnosed with a mental disorder, the YLS/CMI 2.0 predicted violent outcomes with a small effect size, AUC = .59, 95% CI [.48, .70] and non-violent, AUC = .64, 95% CI [.53, .75], and any recidivism outcomes, AUC = .64, 95% CI [.52, .75] with moderate effect sizes. A meta-analytic study by Olver et al. (2014) found a small overall effect for predicting general recidivism, *r_fixed_* = .25, 95% CI [.24, .27]; *k* = 30, although a moderate effect was found when using Canadian samples, *r_fixed_* = .33, 95% CI [.29, .37]; *k* = 12. The YLS/CMI predicted violent recidivism with a small effect size, *r_fixed_* = .22, 95% CI [ .18, .25]; *k* = 13.

## Discussion

Using a standardized, validated risk tool to evaluate youths who have engaged in sexually abusive behaviors, whether they are involved in the forensic mental health system or the juvenile care system is a necessary part of the overarching principles of risk, need, and responsivity, particularly if our overall goal is to reduce further involvement of youths in justice settings. As noted by others (Olver & Stockdale, 2019), risk assessments can inform decisions at various points during the processing of justice-involved youths, such as whether the youth could safely be returned to their community under supervision, the length and type of sentence that should be given, and criminogenic areas that should be targeted in both rehabilitation and supervision. However, the choice of tools has grown over the past couple of decades, and they are varied in their nature, administration, and composition. Although the J-SOAP-II and the ERASOR 2.0 may have been more frequently used in North America prior to 2010 (McGrath et al., 2010), there have been no updates regarding the prevalence of tool use for assessing sexual violence risk among youths. Furthermore, several newly developed tools have been published since the survey was conducted (e.g., MEGA♪, J-RAT, PROFESOR). The rationale for using a particular tool over another varies by setting or individual (see Jung et al., 2013, for discussion), and tool selection may also be contingent on the organization’s policies and requirements; hence, it is an important exercise for practitioners to determine considerations that may be unique to their context, and if tools are pre-selected, to understand the limitations of the risk tool that they employ.

What becomes critical to deliberate are the caveats and challenges when conducting a risk assessment, regardless of which tool is chosen. First, one must be cognizant that risk evaluations, in general, have temporal limits to their validity, and for youths, this ‘shelf-life’ may be even further shortened (Viljoen et al., 2020). Circumstances for youths are dynamic, and their development is ongoing, so reassessment of risk should expectedly be more frequent than risk assessments completed on adults who sexually offend.

Another caveat is the use of risk tools to determine relative and absolute risk since most youths with sexual offence convictions do not continue to reoffend and therefore have low base rates for sexual recidivism (i.e., 4.92% reported in Caldwell, 2016). When youths score high on a risk assessment, it is not necessarily the case that the individual will go on to reoffend sexually. Although some tools (i.e., actuarial) predict adequately for male adolescents, it is important to remember that the outcomes are not the same across all types of sexual reoffences; for example, data seems to be limited with regards to some risk tools predicting sexual recidivism for youths who offend against siblings (Newlands et al., 2019), which can be limiting given that in some samples, sibling abuse is common (Seto & Lalumière, 2010). Similarly, some tools may predict better for youths with a history of sexual offending than those who have a history of general offending (e.g., Rajlic & Gretton, 2010). Fanniff and Letourneau (2012) raised another issue in their examination of the J-SOAP-II regarding times when there is mixed evidence for a risk tool (e.g., in this review, the JRAS had mixed support). They specifically recommend that evaluators do not base significant decisions on the tool. Consequently, it may be best advised that risk tools should not be used as decision-makers for restrictive policies (e.g., institutionalization, registries, notifications) that have long-lasting impact when it comes to youths who have been convicted of sexual offending. However, it is recommended that these tools be used for short-term risk decisions, and it should be acknowledged that risk is fluid and changing and re-evaluations should happen frequently and with regularity. The best use of risk tool for youths is for determining the level of service, identifying intervention targets, and linking recommendations to assessed risk, needs, and responsivity factors.

Our review did not address the application of these risk tools to racial minorities or Indigenous groups in light of the lack of empirical data available. Although there has been a growth of research on adults who are at risk or engaged in sexual abuse (see Shepherd & Lewis-Fernandez, 2016, for discussion; see Lee et al., 2020, for empirical example), this has yet to be examined in any depth with youths. Therefore, a cautious approach to assessing minority and/or Indigenous youths should be taken to reduce bias and unfairness in assessing risk, particularly in justice settings. Vincent and Viljoen (2020) make several recommendations, which include avoiding making score-based classifications of risk, focusing on dynamic risk factors that would be meaningful for mitigating risk, and seeking experience in cultural competence to gain awareness of potential biases.

A final caveat of assessing risk among youths at risk and/or who engaged in sexually abusive behaviors is the limitations of existing research. Notably, that current examinations of risk factors for sexual recidivism in youths are certainly not exhaustive and we may be missing many criminogenic needs that are not included in existing tools (e.g., lack of sleep, Clinkinbeard et al., 2011). Furthermore, we may not be capturing all incidents of sexually abusive behavior. It is already known that youths reoffend at remarkably lower rates than their adult counterparts (Caldwell, 2016); however, given that most youth-perpetrated incidents involve family members, it is a greater likelihood that these incidents are less reported and therefore limits the predictive validity analyses needed to evaluate these tools. Another challenge is the importance and yet limited data on protective factors. In light of the developing youth, it makes sense to build on existing strengths, protective factors, and prosocial goals. However, the research is not strong and much more research is needed to examine their relationship to criminogenic needs and whether they add predictive accuracy over and above other static risk factors (see Viljoen et al., 2020, for discussion). Given that this research is ever-evolving, it would be remiss not to continually re-examine our knowledge of relevant criminogenic needs and risk factors associated with sexual offending among youths.

This review is not without limitations. It is important to note that new tools are constantly being developed, and many have not been published in peer-reviewed journals. Also, the training resources noted in this review are by no means exhaustive. There may be other training offered by the developers of the tool or approved trainers in either specialized workshops or through regional or international conferences.

### Conclusion

It still remains the practitioner’s responsibility to use a defensible approach to risk assessment. This would most reasonably involve employing an objective measure to determine how much service to administer and identify areas that should be targeted in treatment and supervision. Difficult decisions must be made regarding the amount of treatment services provided, as there are practical constraints to resources, and not all youths may need or should require the same quantity of attention. In fact, treatment services provided to low-risk youths should be kept to a minimum, as intensive treatment to low-risk youths is not only an inefficient use of resources, but it may even increase their chances of re-offending (see Lovins et al., 2009). There remain many caveats to overcome in the general field of assessing risk (Helmus, 2018), and with regards to youths specifically, Viljoen et al. (2020) highlight that we are still striving to achieve a culturally‐informed, developmentally‐informed, and gender‐appropriate approach to assessing risk among youths. We have a number of tools at our disposal, but it is necessary for us to be aware of these resources and stay abreast of the challenges in their uses.

## References

[r1] Aebi, M., Plattner, B., Steinhausen, H. C., & Bessler, C. (2011). Predicting sexual and nonsexual recidivism in a consecutive sample of juveniles convicted of sexual offences. Sexual Abuse, 23(4), 456–473. 10.1177/1079063210384634 21406605

[r2] Anderson, V. R., Davidson, W. S., II, Barnes, A. R., Campbell, C. A., Petersen, J. L., & Onifade, E. (2016). The differential predictive validity of the Youth Level of Service/Case Management Inventory: The role of gender. Psychology, Crime & Law, 22(7), 666–677. 10.1080/1068316X.2016.1174861

[r3] Barra, S., Bessler, C., Landolt, M. A., & Aebi, M. (2018). Testing the validity of criminal risk assessment tools in sexually abusive youth. Psychological Assessment, 30(11), 1430–1443. 10.1037/pas0000590 29792506

[r4] Bhanwer, A. (2016). *The Structured Assessment of Protective Factors for Violence Risk – Youth Version (SAPROF-YV): The association between protective factors and aggression in adolescents.* Simon Fraser University, Burnaby, British Columbia, Canada.

[r5] Bonta, J., & Andrews, D. A. (2017). *The psychology of criminal conduct* (6th ed.). Routledge, Taylor & Francis Group.

[r6] Borum, R., Bartel, P., & Forth, A. (2006). *Manual for the Structured Assessment for Violence Risk in Youth (SAVRY).* Odessa, FL, USA: Psychological Assessment Resources.

[r7] Brogan, L., Haney-Caron, E., NeMoyer, A., & DeMatteo, D. (2015). Applying the Risk-Needs-Responsivity (RNR) model to juvenile justice. Criminal Justice Review, 40(3), 277–302. 10.1177/0734016814567312

[r8] Caldwell, M. F. (2016). Quantifying the decline in juvenile sexual recidivism rates. Psychology, Public Policy, and Law, 22(4), 414–426. 10.1037/law0000094

[r9] Caldwell, M. F., Ziemke, M. H., & Vitacco, M. J. (2008). An examination of the sex offender registration and notification act as applied to juveniles: Evaluating the ability to predict sexual recidivism. Psychology, Public Policy, and Law, 14(2), 89–114. 10.1037/a0013241

[r10] Chaffin, M., Berliner, L., Block, R., Johnson, T. C., Friedrich, W., Louis, D. G., Lyon, T., Page, I., Prescott, D., & Silovsky, J. (2006). *Report of the ATSA task force on children with sexual behavior problems*. Association for the Treatment of Sexual Abusers. https://www.atsa.com/pdfs/Report-TFCSBP.pdf 10.1177/1077559507306718 18408214

[r11] Chu, C. M., Ng, K., Fong, J., & Teoh, J. (2012). Assessing youth who sexually offended: The predictive validity of the ERASOR, J-SOAP-II, and YLS/CMI in a non-Western context. Sexual Abuse, 24(2), 153–174. 10.1177/1079063211404250 21825111 PMC4449365

[r12] Clinkinbeard, S. S., Simi, P., Evans, M. K., & Anderson, A. L. (2011). Sleep and delinquency: Does the amount of sleep matter? Journal of Youth and Adolescence, 40(7), 916–930. 10.1007/s10964-010-9594-6 20936500

[r13] Curwen, T. (2011). A framework to assist in evaluating children’s risk to repeat concerning sexual behaviour. In M. Calder (Ed.), *Contemporary practice with young people who sexually abuse: Evidence-based developments* (pp. 263–291). Russell House Publishing, Lyme Regis: Dorset.

[r14] DeLago, C., Schroeder, C. M., Cooper, B., Deblinger, E., Dudek, E., Yu, R., & Finkel, M. A. (2020). Children who engaged in interpersonal problematic sexual behaviors. Child Abuse & Neglect, 105(1), 104260. 10.1016/j.chiabu.2019.104260 31776010

[r15] de Vries Robbé, M., Geers, M., Stapel, M., Hilterman, E., & de Vogel, V., (2015). *SAPROF Youth Version: Guidelines for the assessment of protective factors for violence risk in juveniles*. Vander Hoeven Kliniek part of De Forensische Zorgspecialisten.

[r16] Dolan, M. C., & Rennie, C. E. (2008). The Structured Assessment of Violence Risk in Youth as a predictor of recidivism in a United Kingdom cohort of adolescent offenders with conduct disorder. Psychological Assessment, 20(1), 35–46. 10.1037/1040-3590.20.1.35 18315397

[r17] Epperson, D. L., & Ralston, C. A. (2015). Development and validation of the Juvenile Sexual Offense Recidivism Risk Assessment Tool-II. Sexual Abuse, 27(6), 529–558. 10.1177/1079063213514452 24492618

[r18] Epperson, D. L., Ralston, C. A., Fowers, D., DeWitt, J., & Gore, K. S. (2006). Actuarial risk assessment with juveniles who sexually offend: Development of the Juvenile Sexual Offense Recidivism Risk Assessment Tool-II (JSORRAT-II). In D. S. Prescott (Ed.), *Risk assessment of youth who have sexually abused* (pp. 118–169). Wood ‘N’ Barnes.

[r19] Fanniff, A. M., & Letourneau, E. J. (2012). Another piece of the puzzle: Psychometric properties of the J-SOAP-II. Sexual Abuse, 24(4), 378–408. 10.1177/1079063211431842 22344780

[r20] Gammelgård, M., Koivisto, A., Eronen, M., & Kaltiala, H. R. (2015). Predictive validity of the structured assessment of violence risk in youth: A 4-year follow-up. Criminal Behaviour and Mental Health, 25(3), 192–206. 10.1002/cbm.1921 25042997

[r21] Griffin, H. L., Beech, A., Print, B., Bradshaw, H., & Quayle, J. (2008). The development and initial testing of the AIM2 framework to assess risk and strengths in young people who sexually offend. Journal of Sexual Aggression, 14(3), 211–225. 10.1080/13552600802366593

[r22] Hanson, R. K. (2009). The psychological assessment of risk for crime and violence. Canadian Psychology, 50(3), 172–182. 10.1037/a0015726

[r23] Harris, G. T., Rice, M. E., & Cormier, C. A. (2002). Prospective replication of the Violence Risk Appraisal Guide in predicting violent recidivism among forensic patients. Law and Human Behavior, 26(4), 377–394. 10.1023/A:1016347320889 12182529

[r24] Hart, S. D., Dougas, K. S., & Guy, L. S. (2016). The structured professional judgement approach to violence risk assessment: Origins, nature, and advances. In D. P. Boer, A. R. Beech, T. Ward, L. A. Craig, M. Rettenberger, L. E. Marshall, & W. L. Marshall (Eds.), *The Wiley handbook on the theories, assessment, and treatment of sexual offending* (pp. 643–666). Wiley Blackwell. 10.1002/9781118574003.wattso030

[r25] Helmus, L. M. (2018). Sex offender risk assessment: Where are we and where are we going? Current Psychiatry Reports, 20(6), 46. 10.1007/s11920-018-0909-8 29779064

[r26] Helmus, L. M., & Babchishin, K. M. (2017). Primer on risk assessment and the statistics used to evaluate its accuracy. Criminal Justice and Behavior, 44(1), 8–25. 10.1177/009385481667889831097844

[r27] Hempel, I., Buck, N., Cima, M., & van Marle, H. (2013). Review of risk assessment instruments for juvenile sex offenders: What is next? International Journal of Offender Therapy and Comparative Criminology, 57(2), 208–228. 10.1177/0306624X11428315 22147101

[r28] Henniker, J., Print, B., & Morrison, T. (2002). An inter-agency assessment framework for young people who sexually abuse: Principles, processes and practicalities. Child Care in Practice, 8(2), 114–126. 10.1080/13575270220148567

[r29] Hiscox, S. P., Witt, P. H., & Haran, S. J. (2007). Juvenile Risk Assessment Scale (JRAS): A predictive validity study. The Journal of Psychiatry & Law, 35(4), 503–539. 10.1177/009318530703500406

[r30] Hoge, R. D., & Andrews, D. A. (2002). *Youth Level of Service/Case Management Inventory (YLS/CMI).* Toronto, Ontario, Canada: Multi-Health Systems.

[r31] Jung, S., Pham, A., & Ennis, L. (2013). Measuring the disparity of categorical risk among various sex offender risk assessment measures. Journal of Forensic Psychiatry & Psychology, 24(3), 353–370. 10.1080/14789949.2013.806567

[r32] Kang, T., Beltrani, A., Manheim, M., Spriggs, S., Nishimura, B., Sinclair, S., Stachniuk, M., Pate, E., Righthand, S., Worling, J. R., & Prentky, R. A. (2019). Development of a risk/treatment needs and progress protocol for juveniles with sex offenses. Translational Issues in Psychological Science, 5(2), 154–169. 10.1037/tps0000191

[r33] Koh, L. L., Day, A., Klettke, B., Daffern, M., & Chu, C. M. (2022). The predictive validity of three youth violence assessment instruments: The SAVRY, VRS-YV, and SAPROF-YV. International Journal of Offender Therapy and Comparative Criminology, 66(2-3), 168–185. 10.1177/0306624X20970887 33167725

[r34] Lee, S. C., Hanson, R. K., & Blais, J. (2020). Predictive accuracy of the Static-99R and Static-2002R risk tools for identifying Indigenous and White individuals at high risk for sexual recidivism in Canada. Canadian Psychology, 61(1), 42–57. 10.1037/cap0000182

[r35] Lodewijks, H. P. B., de Ruiter, C., & Doreleijers, T. A. H. (2008). Gender differences in violent outcome and risk assessment in adolescent offenders after residential treatment. International Journal of Forensic Mental Health, 7(2), 133–146. 10.1080/14999013.2008.9914410

[r36] Logan, C. (2016). Structured professional judgement: Applications to sexual offender risk assessment and management. In A. Phenix & H. M. Hoberman (Eds.), *Sexual offending: Predisposing antecedents, assessments and management* (pp. 571–588). Springer Science + Business Media. 10.1007/978-1-4939-2416-5_26

[r37] Lovins, B., Lowenkamp, C. T., & Latessa, E. J. (2009). Applying the risk principle to sex offenders: Can treatment make some sex offenders worse? The Prison Journal, 89(3), 344–357. 10.1177/0032885509339509

[r38] Mann, R. E., Hanson, R. K., & Thornton, D. (2010). Assessing risk for sexual recidivism: Some proposals on the nature of psychologically meaningful risk factors. Sexual Abuse, 22(2), 191–217. 10.1177/1079063210366039 20363981

[r39] Martinez, R., Flores, J., & Rosenfeld, B. (2007). Validity of the Juvenile Sex Offender Assessment Protocol-II (J-SOAP-II) in a sample of urban minority youth. Criminal Justice and Behavior, 34(10), 1284–1295. 10.1177/0093854807301791

[r40] McGrath, R. J., Cumming, G. F., Burchard, B. L., Zeoli, S., & Ellerby, L. (2010). *Current practices and emerging trends in sexual abuser management: The Safer Society 2009 North American survey.* Safer Society Press.

[r41] Meyers, J. R., & Schmidt, F. (2008). Predictive validity of the Structured Assessment for Violence Risk in Youth (SAVRY) with juvenile offenders. Criminal Justice and Behavior, 35(3), 344–355. 10.1177/0093854807311972

[r42] Miccio-Fonseca, L. C. (2012). *Multiplex empirically guided inventory of ecological aggregates for assessing sexually abusive children and adolescents (ages 19 and under)—MEGA♪.* San Diego, CA, USA: Author.

[r43] Miccio-Fonseca, L. C. (2013). MEGA♪: A new paradigm in risk assessment tools for sexually abusive youth. Journal of Family Violence, 28(6), 623–634. 10.1007/s10896-013-9527-8

[r44] Miccio-Fonseca, L. C. (2016). MEGA♪ cross-validation findings on sexually abusive females: Implications for risk assessment and clinical practice. Journal of Family Violence, 31(7), 903–911. 10.1007/s10896-016-9845-8

[r45] Miccio-Fonseca, L. C. (2020). Contemporary risk assessment tools: Should we use them for sexually abusive children ages 4 to 12 years? Journal of Child & Adolescent Trauma, 13(2), 141–151. 10.1007/s40653-019-00267-z 32549926 PMC7289911

[r46] Miccio-Fonseca, L. C. (2021). A critical assessment of the Youth Needs and Progress scale. Journal of Child Sexual Abuse, 30(7), 765–784. 10.1080/10538712.2021.1914260 33899696

[r47] Miller, H. A., & Marshall, E. A. (2019). Comparing solo- and co-offending female sex offenders on variables of pathology, offense characteristics, and recidivism. Sexual Abuse, 31(8), 972–990. 10.1177/1079063218791179 30079820

[r48] Murphy, W. D., Page, I. J., & Hoberman, H. M. (2016). Adolescents who have engaged in sexually abusive behavior: An overview. In A. Phenix & H. M. Hoberman (Eds.), *Sexual offending: Predisposing antecedents, assessments and management* (pp. 185–212). Springer Science + Business Media. 10.1007/978-1-4939-2416-5_9

[r49] Newlands, R., Bennett, N., & O’Donohue, W. (2019). Juvenile Sex Offender Recidivism Risk Assessment Tool-II (JSORRAT-II). In R. D. Morgan (Ed.), *The SAGE encyclopedia of criminal psychology* (pp. 748–750). 10.4135/9781483392240.n243

[r50] Olver, M. E., & Stockdale, K. C. (2019). Criminal risk assessment, juvenile offending. In R. D. Morgan (Ed.), *The SAGE encyclopedia of criminal psychology* (pp. 291–293). 10.4135/9781483392240.n101

[r51] Olver, M. E., Stockdale, K. C., & Wormith, J. S. (2014). Thirty years of research on the level of service scales: A meta-analytic examination of predictive accuracy and sources of variability. Psychological Assessment, 26(1), 156–176. 10.1037/a0035080 24274046

[r52] Onifade, E., Davidson, W., & Campbell, C. (2009). Risk assessment: The predictive validity of the Youth Level of Service Case Management Inventory with African Americans and girls. Journal of Ethnicity in Criminal Justice, 7(3), 205–221. 10.1080/15377930903143544

[r53] Prentky, R., & Righthand, S. (2003). *Juvenile Sex Offender Assessment Protocol II (J-SOAP-II) manual.* U.S. Department of Justice, Office of Justice Programs, Office of Juvenile Justice and Delinquency Prevention. https://www.ojp.gov/pdffiles1/ojjdp/202316.pdf

[r54] Prentky, R., Righthand, S., Worling, J., & Kang, T. (2020). *Final report: Development and implementation project for the Youth Needs and Progress Scale (YNPS).* U. S. Department of Justice. https://www.ojp.gov/pdffiles1/smart/grants/254814.pdf

[r55] Rajlic, G., & Gretton, H. M. (2010). An examination of two sexual recidivism risk measures in adolescent offenders: The moderating effect of offender type. Criminal Justice and Behavior, 37(10), 1066–1085. 10.1177/0093854810376354

[r56] Ralston, C. A., Epperson, D. L., & Edwards, S. R. (2016). Cross-Validation of the JSORRAT-II in Iowa. Sexual Abuse, 28(6), 534–554. 10.1177/1079063214548074 25179400

[r57] Rasmussen, L. A. L. (2018). Comparing predictive validity of JSORRAT-II and MEGA♪ with sexually abusive youth in long-term residential custody. International Journal of Offender Therapy and Comparative Criminology, 62(10), 2937–2953. 10.1177/0306624X17726550 28863722

[r58] Rasmussen, L. A. L., & Fagundes, M. (2022). A critique of the *Youth Needs and Progress Scale* and its applicability to individuals ages 18 to 25 with sexually abusive behavior. Journal of Aggression, Maltreatment & Trauma, 31(6), 734–752. 10.1080/10926771.2021.1983682

[r59] Rennie, C. E., & Dolan, M. C. (2010). Predictive validity of the youth level of service/case management inventory in custody sample in England. Journal of Forensic Psychiatry & Psychology, 21(3), 407–425. 10.1080/14789940903452311

[r60] Rice, M. E., & Harris, G. T. (2005). Comparing effect sizes in follow-up studies: ROC area, Cohen’s *d,* and *r.* Law and Human Behavior, 29(5), 615–620. 10.1007/s10979-005-6832-7 16254746

[r61] Rich, P. (2017a, November 5). *J-RAT: Juvenile Risk Assessment Tool, version 4*. Retrieved June 16, 2021 from http://www.philrich.net/risk-assessment-instruments.html

[r62] Rich, P. (2017b, November 5). *LA-SAAT: Latency Age-Sexual Adjustment and Assessment Tool, version 4*. Retrieved June 16, 2021 from http://www.philrich.net/risk-assessment-instruments.html

[r63] Rich, P. (2019, July 23). *ID/J-RAT*: Intellectual Disability Juvenile Risk Assessment Tool, version 5.* Retrieved June 16, 2021 from http://www.philrich.net/risk-assessment-instruments.html

[r64] Righthand, S., Worling, J. R., Prentky, R. A., & Kang, T. (2020). *Youth Needs and Progress Scale & User Guide.* Fairleigh Dickinson University. https://www.ncsby.org/sites/default/files/Youth%20Needs%20and%20Progress%20Scale-%20July%207,%202020.pdf

[r65] Seto, M. C., & Lalumière, M. L. (2010). What is so special about male adolescent sexual offending? A review and test of explanations through meta-analysis. Psychological Bulletin, 136(4), 526–575. 10.1037/a0019700 20565168

[r66] Shepherd, S. M., & Lewis-Fernandez, R. (2016). Forensic risk assessment and cultural diversity: Contemporary challenges and future directions. Psychology, Public Policy, and Law, 22(4), 427–438. 10.1037/law0000102

[r67] Sijtsema, J. J., Kretschmer, T., & van Os, T. (2015). The Structured Assessment of Violence Risk in Youth in a large community sample of young adult males and females: The TRAILS study. Psychological Assessment, 27(2), 669–677. 10.1037/a0038520 25558967

[r68] Stockdale, K. C., Olver, M. E., & Wong, S. C. P. (2014). The validity and reliability of the Violence Risk Scale–Youth Version in a diverse sample of violent young offenders. Criminal Justice and Behavior, 41(1), 114–138. 10.1177/0093854813496999

[r69] ter Beek, E., van der Rijken, R. E. A., Kuiper, C. H. Z., Hendriks, J., & Stams, G. J. J. M. (2018). The allocation of sexually transgressive juveniles to intensive specialized treatment: An assessment of the application of RNR principles. International Journal of Offender Therapy and Comparative Criminology, 62(5), 1179–1200. 10.1177/0306624X16674684 27913713

[r70] Vess, J. (2011). Risk assessment with female sex offenders: Can women meet the criteria of community protection laws? Journal of Sexual Aggression, 17(1), 77–91. 10.1080/13552600.2010.528844

[r71] Viljoen, J. L., Cochrane, D. M., & Jonnson, M. R. (2018). Do risk assessment tools help manage and reduce risk of violence and reoffending? A systematic review. Law and Human Behavior, 42(3), 181–214. 10.1037/lhb0000280 29648841

[r72] Viljoen, J. L., Jonnson, M. R., & Shepherd, S. M. (2020). Assessing risk for violent, general, and sexual offending in adolescents: Recent advances and future directions. In J. S. Wormith, L. A. Craig, & T. E. Hogue (Eds.), *The Wiley handbook of what works in violence risk management: Theory, research, and practice* (pp. 223–249). Wiley Blackwell. 10.1002/9781119315933.ch11

[r73] Viljoen, J. L., Mordell, S., & Beneteau, J. L. (2012). Prediction of adolescent sexual reoffending: A meta-analysis of the J-SOAP-II, ERASOR, J-SORRAT-II, and Static-99. Law and Human Behavior, 36(5), 423–438. 10.1037/h0093938 22353046

[r74] Viljoen, J. L., Scalora, M., Cuadra, L., Bader, S., Chávez, V., Ullman, D., & Lawrence, L. (2008). Assessing risk for violence in adolescents who have sexually offended: A comparison of the J-SOAP-II, J-SORRAT-II, and SAVRY. Criminal Justice and Behavior, 35(1), 5–23. 10.1177/0093854807307521

[r75] Viljoen, J. L., Shaffer, C. S., Muir, N. M., Cochrane, D. M., & Brodersen, E. M. (2019). Improving case plans and interventions for adolescents on probation: The implementation of the SAVRY and a structured case planning form. Criminal Justice and Behavior, 46(1), 42–62. 10.1177/0093854818799379

[r76] Vincent, G. M., & Viljoen, J. L. (2020). Racist algorithms or systemic problems? Risk assessments and racial disparities. Criminal Justice and Behavior, 47(12), 1576–1584. 10.1177/0093854820954501

[r77] Wong, S. C. P., & Gordon, A. (1999–2003). *Violence risk scale.* Department of Psychology, University of Saskatchewan. http://www.psynergy.ca

[r78] Wong, S. C. P., Lewis, K., Stockdale, K., & Gordon, A. (2004-2011). *The Violence Risk Scale-Youth Version.* Unpublished manuscript. University of Saskatchewan, Saskatoon, Saskatchewan, Canada.

[r79] Worling, J. R. (2017, June 14). *PROFESOR: Protective + risk observations for eliminating sexual offense recidivism.* http://www.drjamesworling.com/profesor.html

[r80] Worling, J. R., Bookalam, D., & Litteljohn, A. (2012). Prospective validity of the Estimate of Risk of Adolescent Sexual Offense Recidivism (ERASOR). Sexual Abuse, 24(3), 203–223. 10.1177/1079063211407080 21969313

[r81] Worling, J. R., & Curwen, T. (2001). *Estimate of Risk of Adolescent Sexual Offense Recidivism, Version 2.0.* Ontario Ministry of Community and Social Services.

[r82] Wylie, L. E., Clinkinbeard, S. S., & Hobbs, A. (2019). The application of risk-needs programming in a juvenile diversion program. Criminal Justice and Behavior, 46(8), 1128–1147. 10.1177/0093854819859045

[r83] Zara, G., Farrington, D. P., Freilone, F., & Losel, F. (2020). Assessment, management, and treatment of sex offenders: What is known, what is controversial, what needs further investigation. Rassegna Italiana di Criminologia, 14(3), 166–185. 10.7347/RIC-032020-p166

[r84] Zeng, G., Chu, C. M., & Lee, Y. (2015). Assessing protective factors of youth who sexually offended in Singapore: Preliminary evidence on the utility of the DASH-13 and the SAPROF. Sexual Abuse, 27(1), 91–108. 10.1177/1079063214561684 25527632 PMC4441883

